# Evolving Horizons in Pediatric Leukemia: Novel Insights, Challenges, and the Journey Ahead

**DOI:** 10.7759/cureus.67480

**Published:** 2024-08-22

**Authors:** Piere R Tito Rodriguez, Deepalee Mehta, Muhammad Subhan, Ratan Pal Yadav, Bibi Sarah Yousofzai, Ebtesam H Al-Najjar, Ruqiya Bibi, Mohamed Idries, Atinder Singh, Muhammad Adnan

**Affiliations:** 1 Medicine, Universidad Privada del Valle, La Paz, BOL; 2 Internal Medicine, Bharati Vidyapeeth Medical College, Sangli, Sangli, IND; 3 Medicine, Allama Iqbal Medical College, Jinnah Hospital, Lahore, PAK; 4 Pediatrics, NRI Academy of Sciences, Guntur, IND; 5 Obstetrics and Gynaecology, Be Team International Cure Hospital, Kabul, AFG; 6 Internal Medicine, Houston Methodist Hospital, Houston, USA; 7 Biochemistry, St. George's University School of Medicine, St. George's, GRD; 8 Medicine, World College of Medical Sciences and Research Hospital, Gurugram, IND; 9 Pediatrics, Lady Reading Hospital, Peshawar, PAK; 10 Pediatrics, Khyber Medical College, Peshawar, PAK

**Keywords:** chemotherapy, immunotherapy, acute myeloid leukemia (aml), acute lymphoblastic leukemia (all), pediatric leukemia

## Abstract

Pediatric leukemia, encompassing acute lymphoblastic leukemia (ALL) and acute myeloid leukemia, remains a formidable challenge despite significant treatment advancements. This review examines recent developments in immunotherapy, chemotherapy, and bone marrow transplantation for pediatric leukemia through a comprehensive analysis of recent literature, focusing on critical studies and clinical trials. Immunotherapy, including monoclonal antibodies, such as blinatumomab and inotuzumab ozogamicin, and chimeric antigen receptor T-cell therapies, such as tisagenlecleucel and brexucabtagene autoleucel, have demonstrated promising results in relapsed or refractory B-cell ALL (B-ALL), achieving notable remission rates with manageable side effects. Chemotherapy continues to be the primary treatment, utilizing multiphase regimens tailored to individual risk profiles. Bone marrow transplantation, especially allogeneic stem cell transplantation, offers potential cures for high-risk or relapsed cases, though it poses risks including graft-versus-host disease and infections. Despite these advancements, treatment resistance, toxicity, and accessibility persist. This review also discusses the long-term outcomes among pediatric leukemia survivors, focusing on late-onset side effects associated with treatments such as chemotherapy and bone marrow transplantation, encompassing secondary malignancies, organ dysfunction, and neurocognitive impacts. Ongoing research and clinical trials are crucial to refine these therapies, enhance their efficacy, and reduce adverse effects, ultimately improving young patients' survival and quality of life.

## Introduction and background

Leukemia, a type of blood cancer, begins in the body's blood-forming tissues, notably the bone marrow and lymphatic system [1]. This disease results from the uncontrolled proliferation of immature or irregular white blood cells, leading to distressing symptoms and complications [2]. Pediatric leukemia, which constitutes approximately 30% of all cancers affecting children below 15 years, is the most prevalent cancer type within this age group [2,3]. Its incidence varies due to several factors: geographical differences can influence environmental and genetic predispositions, age and gender play a role as certain types are more prevalent in specific age groups and more common in boys, and ethnic disparities affect genetic susceptibility, leading to variations in disease rates across different populations [4]. The International Agency for Research on Cancer reported a global incidence rate of 4.8 cases per 100,000 children aged 0-14, with age standardization considered in 2020 [5]. This rate exhibits variations, with the highest incidence observed in North America, Europe, and Oceania and the lowest in Africa and Asia [5,6]. Notably, leukemia is more frequently diagnosed in boys than girls, with a male-to-female ratio of 1.3:1 [7].

Additionally, White children are more prevalent than their Black or Asian counterparts [8]. Leukemia can be classified into several subtypes, depending on the specific type of white blood cell involved and the speed of disease progression [9]. The predominant subtypes in pediatric cases are acute lymphoblastic leukemia (ALL) and acute myeloid leukemia (AML), constituting approximately 75% and 20% of diagnoses, respectively [10]. ALL predominantly affects immature lymphocytes, a type of white blood cell vital for infection defense [11]. Recently, the survival rate for children aged 0-14 years with ALL has surpassed 90% in many high-income countries, marking a substantial improvement compared to half a century ago when ALL was considered incurable [10,11]. AML, on the other hand, affects immature myeloid cells that can be distinguished into various blood cell types, including red blood cells, platelets, and granulocytes [11-13].

The journey of leukemia treatment has traversed a fascinating path, evolving from early descriptions in the 19th century to the introduction of chemotherapy by Sidney Farber in the 20th century, leading to the concept of combination chemotherapy by Jean-Bernard and curative protocols by Donald Pinkel [14]. Bone marrow transplantation (BMT) and, more recently, immunotherapy have marked significant milestones in leukemia treatment, with BMT particularly crucial in high-risk or relapsed cases, offering potential cures despite associated risks such as graft-versus-host disease (GVHD) and infections [15]. Genetic anomalies play a pivotal role in pediatric leukemia, including chromosomal translocations, deletions, duplications, inversions, and mutations affecting genes associated with cell growth, differentiation, apoptosis, and DNA repair [4,15,16]. Molecular mechanisms contributing to pediatric leukemia encompass aberrant signaling pathways, epigenetic changes, microRNA dysregulation, and interactions within the tumor microenvironment, all affecting cell proliferation, survival, differentiation, and migration [15-18]. Environmental factors that might influence leukemia risk include exposure to ionizing radiation, chemical carcinogens, infectious agents, and immunological factors, although the conclusive evidence supporting these associations varies [19].

Common symptoms encompass fatigue, pallor, fever, bleeding, bruising, bone pain, lymph node enlargement, hepatosplenomegaly, and weight loss [3]. Leukemia can also affect other organ systems, including the central nervous system (CNS), skin, eyes, lungs, kidneys, heart, or testes [7,8]. Diagnosing pediatric leukemia necessitates a comprehensive approach, encompassing a detailed patient history, physical examination, laboratory tests, imaging scans, and bone marrow aspiration or biopsy [18,19]. Laboratory assessments include blood completion, smears, blood chemistry, coagulation profiling, immunophenotyping, cytogenetic examination, and molecular analysis [18,19]. Diagnostic imaging (e.g., X-rays, computed tomography scans, magnetic resonance imaging, positron emission tomography scans) evaluates organ conditions [19,20]. Bone marrow aspiration and biopsy confirm the presence of leukemia, its subtype, and disease risk [19,20].

Management of pediatric leukemia is based on the subtype, risk group, response, and complications [18-20]. Chemotherapy is the primary treatment, with different phases, to kill leukemic cells and prevent relapse [19]. BMT is another option for some patients with high-risk or relapsed disease [5,16-18,20]. Immunotherapy harnesses the body's immune system to identify and eliminate cancerous cells [20-22]. It has shown promising results in some patients but also has challenges [21,22]. This review covers pediatric leukemia's history, causes, features, and treatments; explores BMT as an alternative treatment; and highlights the recent breakthroughs and challenges in immunotherapy and chemotherapy. Its ultimate goal is to serve as a valuable resource for healthcare professionals and the scientific community, enhancing the understanding of pediatric leukemia and the evolving treatment landscape.

## Review

Leukemia, a complex and multifaceted group of hematologic malignancies, continues to pose significant challenges in pediatric and adult populations [1-4]. Recent advancements in understanding the genetic and molecular underpinnings of these diseases have paved the way for innovative therapeutic approaches [4-7]. This discussion delves into the intricate genetic heterogeneity and pathogenesis of AML, explores the evolving landscape of targeted therapies and immunotherapy, and highlights the strides made in treating pediatric ALL [4-7]. Furthermore, it examines the advancements in chemotherapy regimens to enhance efficacy and manage relapse in pediatric leukemia [8-11]. It underscores the critical role of BMT, addressing the associated challenges and recent advances [11,12]. The treatment strategies for AML and ALL differ significantly due to their distinct biological and clinical characteristics [1-11]. Table 1 outlines the primary goals, therapeutic approaches, and critical aspects of managing AML and ALL [1-11].

**Table 1:** Comparison of treatment strategies for AML and ALL. AML: acute myeloid leukemia; ALL: acute lymphoblastic leukemia; MRD: minimal residual disease; NPM1: nucleophosmin 1; FLT3: Fms-like tyrosine kinase-3; CNS: central nervous system; SCT: Stem cell transplantation; CR: complete remission.

Aspect	AML	ALL
Primary goal	Achieve CR by eliminating leukemic blasts	Achieve CR by eliminating leukemic lymphoblasts
Induction therapy	- Standard 7+3 regimen (cytarabine for seven days, anthracycline for three days)	- Multi-drug regimen (e.g., vincristine, prednisone, doxorubicin, asparaginase)
Consolidation therapy	- High-dose cytarabine or stem cell transplant	- Continued multi-drug regimen or high-dose methotrexate and cytarabine
Maintenance therapy	- Not commonly used	- Typically includes methotrexate, 6-mercaptopurine, vincristine, and prednisone
Common drugs	- Cytarabine, daunorubicin, idarubicin, mitoxantrone	- Vincristine, doxorubicin, cyclophosphamide, asparaginase, methotrexate, cytarabine
Targeted therapy	- FLT3 inhibitors (e.g., midostaurin, gilteritinib)	- Tyrosine kinase inhibitors (e.g., imatinib, dasatinib for Ph+ ALL)
SCT	- Used in high-risk or relapsed patients	- Used in high-risk or relapsed patients
Response monitoring	- Bone marrow biopsy, molecular markers (e.g., NPM1, FLT3)	- Bone marrow biopsy, MRD assessment
Prophylaxis	- Not routinely required	- CNS prophylaxis with intrathecal methotrexate/cytarabine
Duration of treatment	- Intensive initial phase (four to six months)	- Longer duration (two to three years, including maintenance phase)

Comprehensive insights into AML: genetic heterogeneity, pathogenesis, and advances in targeted therapies and immunotherapy

AML is a complex and diverse hematologic malignancy marked by the clonal proliferation of myeloid cells [1]. AML is known for its heterogeneity, presenting various subtypes based on genetic abnormalities and blast count [1,2]. The disease can manifest in numerous symptoms, including fatigue, fever, easy bruising, and increased infection susceptibility [3,4].

Recent advancements in genomic profiling have unveiled a plethora of mutations and chromosomal alterations that not only aid in the stratification of AML subtypes but also influence prognosis and therapeutic responses [4-7]. AML begins from hematopoietic stem and progenitor cells acquiring somatic mutations, which grant these cells the ability to self-renew and proliferate abnormally. Common early mutations, including DNMT3A, TET2, and ASXL1, often found in clonal hematopoiesis, a pre-malignant state [5,6]. Mutations, such as FLT3-ITD (Fms-like tyrosine kinase-3 internal tandem duplication), nucleophosmin 1 (NPM1), and RUNX1-RUNX1T1 (RUNX1 translocation partner 1), play pivotal roles in the pathogenesis and clinical outcomes of AML [5,6]. FLT3-ITD mutations, characterized by ITDs in the *FLT3* gene, are associated with poor prognosis and increased risk of relapse due to heightened cell proliferation and resistance to chemotherapy [6,7]. NPM1 mutations involving nucleophosmin are one of the most frequent genetic alterations in AML and are associated with a favorable prognosis, often predicting better response to standard chemotherapy [6,7]. The RUNX1-RUNX1T1 fusion gene, resulting from t(8;21) translocation, is characteristic of AML with favorable outcomes, affecting myeloid differentiation and response to treatment [6,7].

Understanding these mutations not only aids in risk stratification but also informs targeted therapy approaches, such as FLT3 inhibitors for FLT3-ITD-positive AML or intensive chemotherapy regimens for NPM1-mutated AML [7,8]. The International Consensus Classification and the updated World Health Organization (WHO) classification emphasize genetic aberrations in defining AML subtypes, with key abnormalities including translocations like t(8;21 (q22;q22.1)/RUNX1::RUNX1T1 and inv(16)(p13.1q22) or t(16;16)(p13.1;q22)/CBFB::MYH11 [5,6]. Genetic abnormalities in AML take precedence in the classification hierarchy, guiding treatment decisions and prognostic assessments [5,6]. Mutations such as FLT3-ITD, NPM1, and RUNX1-RUNX1T1, among others, play critical roles in risk stratification and therapeutic approaches [7,8]. AML treatment typically involves intensive chemotherapy regimens aimed at achieving complete remission (CR), followed by consolidation therapy with or without allogeneic BMT for eligible candidates [7,8]. Therapy-related AML or AML evolving from prior myelodysplastic syndrome (MDS) represents distinct clinical entities necessitating tailored management strategies, often incorporating targeted therapies or novel agents based on genetic profiles [5,6]. In contrast, ALL treatment frequently includes prolonged maintenance therapy following induction and consolidation phases, aiming to eradicate residual disease and prevent relapse [5-8]. Recent advancements in ALL therapy have introduced immunotherapeutic approaches such as monoclonal antibodies (mAbs) (e.g., blinatumomab, inotuzumab ozogamicin) and chimeric antigen receptor (CAR) T-cell therapies (e.g., tisagenlecleucel, brexucabtagene autoleucel), targeting CD19-expressing B cells in relapsed or refractory cases [5-8]. These therapies offer promising outcomes with manageable side effects, reshaping treatment paradigms for ALL towards more targeted and less toxic interventions [5-8].

Advancements in genetic profiling have led to the growth of targeted therapies, such as FLT3 inhibitors (e.g., midostaurin, gilteritinib) and IDH inhibitors (e.g., ivosidenib, enasidenib), which have shown efficacy in treating AML with specific mutations [5,6]. Prognostic markers, including mutations in genes like *NPM1* and *CEBPA*, are associated with better prognosis, while others like TP53 and complex karyotypes indicate poorer outcomes, guiding therapeutic decisions [6].

Core-binding factor AML (CBF-AML), characterized by translocations involving *RUNX1* and *CBFB* genes, is generally associated with a favorable prognosis and reasonable response to high-dose cytarabine post-induction therapy [6]. FLT3-mutated AML, with FLT3-ITD and FLT3-TKD mutations, leads to aggressive disease and poor outcomes, although FLT3 inhibitors have significantly improved survival rates despite resistance challenges [7,8].

TP53-mutated AML is associated with complex karyotypes and inferior prognosis, with emerging therapies like APR-246 aiming to restore p53 function, offering potential treatment avenues [7,8]. Therapy-related AML (t-AML), developing as a secondary malignancy following chemotherapy or radiation therapy (RT) for other cancers, often presents with adverse cytogenetic features and poor outcomes, necessitating aggressive treatment approaches [7,8].

Acute promyelocytic leukemia (APL), characterized by the t(15;17) translocation leading to the PML-RARA fusion gene, has transformed into a highly curable form of AML with treatment using all-trans retinoic acid (ATRA) and arsenic trioxide (ATO) [1,2]. The standard treatment regimen for AML often includes chemotherapy and hematopoietic stem cell transplantation (SCT), which has been achieved with inconsistent grades of victory [6,7]. Resistance to treatment and relapse remain significant hurdles, highlighting the need for a deeper exploration into the mechanisms of drug resistance and disease recurrence [6,7].

In the 1970s, the "7+3 regimen" comprising cytarabine and an anthracycline emerged as the standard induction therapy for AML [6,7]. Following induction therapy in AML, the role of maintenance therapy varies based on risk stratification and treatment response [7,8]. For patients with favorable-risk AML, such as those with CBF-AML or NPM1 mutations without high allelic burden FLT3-ITD mutations, consolidative therapy often involves high-dose cytarabine to reduce the risk of relapse and improve outcomes [5,6]. Maintenance therapy post-induction aims to eradicate residual disease and sustain remission, typically involving less intensive chemotherapy or targeted agents tailored to individual genetic profiles [5,6]. In contrast, patients with adverse-risk or intermediate-risk AML face higher relapse rates despite induction therapy, prompting consideration for allogeneic hematopoietic SCT [6,7]. Post-transplant maintenance therapy prevents graft rejection, manages GVHD, and supports long-term remission [7,8]. Strategies may include immunosuppressive agents, targeted therapies to maintain minimal residual disease (MRD)-negative status, or vaccination protocols to boost immune surveillance against leukemia relapse [9,10]. Outcomes are challenging for older patients due to poor tolerance to intensive chemotherapy and a higher prevalence of adverse cytogenetics and mutations [11,12]. Historically, treatment options for older, frailer patients included low-dose cytarabine (LDAC) or hypomethylating agents (HMAs) such as azacitidine and decitabine, with limited survival benefits [11,12].

Mutation-specific targeted therapies have significantly advanced the treatment landscape for AML [4-8]. Mutations in FLT3 are present in about one-third of newly diagnosed AML cases [7,8]. Over the last 15 years, several FLT3 inhibitors have been developed and tested in clinical trials [8,9]. First-generation FLT3 inhibitors such as midostaurin and sorafenib have broad kinome profiles, whereas second-generation inhibitors such as quizartinib and crenolanib are more FLT3-specific [10,11]. Quizartinib, a potent type II FLT3 inhibitor, has shown efficacy in relapsed/refractory FLT3-mutated AML, with notable responses even in FLT3-wild-type disease [11,12]. Gilteritinib, a type I inhibitor targeting AXL, has demonstrated effectiveness as a single therapy in relapsed/refractory FLT3-mutated AML and significantly improved survival compared to salvage chemotherapy [11,12]. However, therapeutic resistance remains challenging, often driven by secondary FLT3 mutations [11,12]. Combining azacitidine with quizartinib has shown promising results in older adults with FLT3-ITD-mutated AML [12,13].

IDH1 and IDH2 inhibitors, such as ivosidenib and enasidenib, have demonstrated efficacy in patients with relapsed or refractory IDH1- or IDH2-mutated AML [4,13]. These inhibitors can induce the differentiation of malignant cells, leading to an isocitrate dehydrogenase (IDH) differentiation syndrome in some patients [13,14]. RAS pathway alterations can confer resistance to FLT3, IDH, and BCL2 inhibitor therapies. MEK inhibitors such as selumetinib and trametinib have shown modest responses in relapsed or refractory RAS-mutated AML, although resistance often develops through compensatory activation of the phosphatidylinositol 3-kinase (PI3K)-protein kinase B (AKT)-mammalian target of rapamycin (mTOR) pathway [15,16].

Other targeted therapies include CBF-AML with KIT mutations and multikinase inhibitors such as midostaurin and dasatinib, which have shown promise when added to standard induction and consolidation therapies [16,17]. TP53 mutations, associated with poor prognosis in AML, present a challenge due to limited therapeutic options [18]. Novel agents like APR-246, which restore p53 function, are investigated with azacitidine to induce apoptosis in TP53-mutated AML or MDS [17,18].

Targeting the apoptotic pathway, which is dysregulated in AML, represents a pivotal strategy to restore normal cell death mechanisms and inhibit leukemic growth [18]. Venetoclax is a second-generation selective BCL2 inhibitor that has revolutionized AML treatment [19]. Studies have demonstrated its efficacy in both relapsed/refractory AML and newly diagnosed AML, particularly when combined with low-intensity therapies like azacitidine, decitabine, or LDAC [18-20]. Despite these advances, resistance mechanisms involving the upregulation of MCL1 remain challenging [18,20]. Preclinical data support the synergy between MCL1 inhibitors and venetoclax, offering a potential strategy to overcome resistance [18-20].

BH3 profiling is emerging as a valuable tool to assess baseline dependency on apoptotic proteins and predict responses to specific BH3 agents, guiding personalized treatment decisions [21]. Advances in mAbs targeting leukemia surface antigens, including bispecific T-cell engagers (BiTEs) and antibody-drug conjugates (ADC), represent innovative strategies to enhance immune-mediated cytotoxicity against AML cells [22-25]. Gemtuzumab ozogamicin (GO) is an ADC that targets CD33, a surface antigen commonly expressed on leukemic cells in AML [22]. GO was reapproved in 2017 for use in first-line therapy, particularly in combination with standard induction regimens and as a single agent for older, unfit patients or those with relapsed/refractory disease [22]. Investigational mAbs targeting various surface markers in AML, such as CD123, CD45, CLL1, TIM3, CD47, and CD70, have shown promise, necessitating further exploration and optimization [23,24]. Immune checkpoint inhibitors involving anti-programmed cell death protein 1 (PD-1), PD-L1, and cytotoxic T-lymphocyte associated protein 4 (CTLA4) antibodies are being investigated in AML [25,26]. Studies combining azacitidine with checkpoint inhibitors have shown promising response rates, particularly in patients without HMA exposure [25,26]. Mechanisms of resistance to these therapies, including upregulation of inhibitory checkpoint proteins like CTLA4, are actively being explored [25,26]. Targeting macrophage checkpoints, such as CD47, which inhibits phagocytosis of AML cells by macrophages, has shown promise with agents like Hu5F9-G4 in combination with azacitidine [26,27].

Advances in the genetic landscape and immunotherapy of pediatric ALL

ALL is a rapidly progressing malignancy characterized by an overproduction of immature lymphoblasts [2]. ALL is the most prevalent pediatric cancer, representing 25% of all pediatric cancers [2,3]. It is characterized by the overproduction of immature lymphocytes, leading to bone marrow failure and systemic disease [2,3]. The genetic landscape of B-ALL in pediatric cases encompasses over 30 distinct subgroups defined by chromosomal abnormalities and gene rearrangements, each carrying significant prognostic and therapeutic implications [28]. Among the low-risk genetic subgroups, ETV6/RUNX1-Rearranged ALL represents approximately 20% of pediatric ALL cases and generally has a favorable outcome; it shares a gene expression profile and immunophenotype with ETV6/RUNX1-rearranged ALL but lacks the fusion gene, with genetic alterations including changes in ETV6, IKZF1, and TCF3, making outcomes appear less favorable compared to ETV6/RUNX1 fusion, necessitating careful monitoring and potentially more aggressive treatment strategies [28]. NUTM1-Rearranged ALL, representing 5-7% of infant ALL cases and 21.7% of non-KMT2A-rearranged infant ALL but rare in older children, involves partners such as ACIN1, CUX1, BRD9, and ZNF618 [29]. Early studies suggest a favorable prognosis with a four-year overall survival (OS) rate of 100%, indicating a potentially less aggressive form of ALL that might allow for treatment de-escalation [29]. Hyperdiploid ALL, the most common subtype, accounting for up to 25% of pediatric ALL cases, responds well to methotrexate treatment, partly due to increased expression of the *SLC19A1* gene involved in folate transport [30]. High-risk genetic subgroups include hypodiploid ALL, defined by fewer than 44 chromosomes and having a poor prognosis with varying survival rates based on specific hypodiploid categories (near haploid, low hypodiploid, or high hypodiploid) [31]. BCR/ABL1-Positive ALL, also known as Philadelphia chromosome-positive ALL, constitutes 2-3% of pediatric ALL cases, with tyrosine kinase inhibitors (TKIs) having markedly improved outcomes for this subgroup [31]. BCR/ABL1-Like ALL shares a similar gene expression profile with BCR/ABL1-positive ALL but lacks the BCR/ABL1 fusion, is associated with worse outcomes, and frequently features JAK-STAT signaling activating mutations or ABL1-class fusions [32]. Intermediate-risk genetic subtypes include TCF3/PBX1-Rearranged ALL, occurring in 2-5% of pediatric ALL cases and linked to a higher incidence of CNS relapse [33]. Intrachromosomal Amplification of Chromosome 21 (iAMP21) is characterized by multiple copies of a region of chromosome 21, including the *RUNX1* gene, and is associated with older age at diagnosis and low white blood cell counts [33].

T-ALL, a subtype of leukemia affecting T-cells that accounts for about 12-15% of pediatric ALL cases, is more prevalent in boys and commonly occurs in patients of African ancestry [34]. It exhibits diverse genetic alterations, including mutations in the *PHF6* gene and abnormal expression of various oncogenes and transcription factors, but lacks a clear genetic classification linked to prognosis [34]. PHF6 mutations on chromosome X are present in 16% of pediatric T-ALL cases [34]. Oncogene expression involves TAL1, TAL2, LYL1, LMO1, LMO2, TLX1, TLX3, and HOXA [34]. NOTCH1 mutations are found in over 70% of T-ALL cases [34]. Approximately 25% of patients harbor JAK-STAT mutations, making them potential candidates for JAK inhibitors [32-34]. Ongoing research focuses on optimizing these therapies to improve their safety profile, reduce the risk of relapse, and extend their application to other hematological malignancies [31-34]. Recent research on germline TP53 variants in low-hypodiploid ALL has revealed significant findings; hypodiploid ALL, a rare and aggressive subtype of childhood ALL, is linked with poor prognosis and often needs intensive therapy [31]. Studies have shown that low-hypodiploid ALL frequently harbors TP53 variants, mostly germline mutations, suggesting a potential link to Li-Fraumeni syndrome, which increases the risk of secondary tumors [31].

An Italian cohort study of hypodiploid pediatric ALL patients diagnosed between 2000 and 2019 found that 50% exhibited TP53 variants, with 19 of 20 cases being low-hypodiploid ALL; of these, 13 were pathogenic, and six were classified as variants of unknown significance (VUS) [30,31]. Notably, 65% of patients had germline TP53 variants, underscoring the importance of genetic counseling and surveillance [31]. The presence of germline TP53 variants significantly affects prognosis and predisposition, with higher risks of relapse and secondary malignancies [31]. Current studies and trials continue to purify our understanding of ALL genetics, advancing precision in treatment protocols to improve survival rates and reduce the risk of relapse across all genetic subtypes of ALL [28-31]. These groundbreaking strategies and collaborative efforts offer new avenues of hope for children and their families amidst the challenging landscape of this devastating disease. As we explore these advancements, it becomes evident that they promise improved outcomes and an enhanced quality of life for young patients grappling with this formidable challenge. The summary of critical studies and their characteristics related to pediatric leukemia management is outlined in Table 2.

**Table 2:** Summary of key studies on pediatric leukemia management. ALL: acute lymphoblastic leukemia; LMICs: low and middle-income countries; AML: acute myeloid leukemia; ADE therapy: cytarabine, daunorubicin hydrochloride, and etoposide phosphate; CML: chronic myeloid leukemia.

Authors	Year of publication	Type of study	Results
Rujkijyanont and Inaba [29]	2024	Review article	Discussed diagnostic and treatment strategies for pediatric ALL in LMICs, highlighting the need for resource-adapted multidisciplinary collaborations
Hoerst et al. [31]	2024	Educational presentation	Described evolving treatment strategies in pediatric leukemia, including the role of trials and managing side effects
Garg et al. [38]	2024	Phase II study	Demonstrated the feasibility and effectiveness of outpatient ADE therapy for pediatric AML relapse
Sembill et al. [39]	2023	Review article	Provided recommendations for managing pediatric CML in the blast phase, including diagnosis, treatment, and monitoring
Brown et al. [28]	2020	Review article	Updated guidelines for pediatric ALL, emphasizing risk-adapted therapy and supportive care to improve survival rates

**Figure 1:**
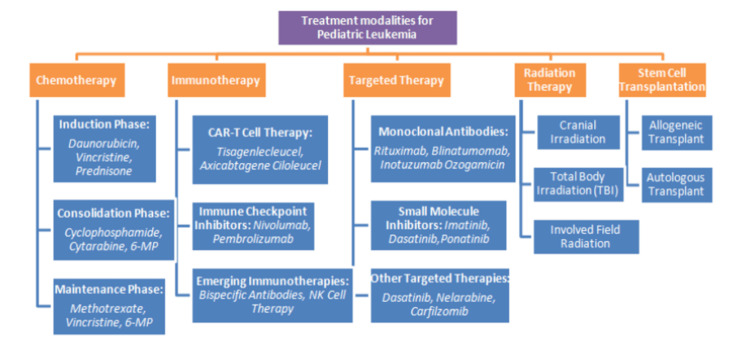
Comprehensive treatment modalities for pediatric leukemia. CAR: chimeric antigen receptor; NK: natural killer.

Figure 1 depicts the varied treatment modalities in ALL, signifying the multifaceted approach addresses this complex condition [1-15, 22-27].

Immunotherapy for ALL includes mAbs, CAR T-cell therapy, and other novel therapies [28-32]. mAbs include blinatumomab and inotuzumab ozogamicin [29]. Blinatumomab is a BiTE that binds to CD19 on B-cell leukemia cells and CD3 on T cells, activating the cytotoxic function of T cells [29]. In a phase III study by Kantarjian et al., blinatumomab showed a CR rate of 43% in adults with relapsed or refractory B-ALL compared to 25% in the chemotherapy group [30]. However, side effects include cytokine release syndrome (CRS) and neurotoxicity, reported in 15% and 25% of patients, respectively [29].

Inotuzumab ozogamicin is an ADC targeting CD22, delivering a cytotoxic agent directly to leukemia cells [30]. In the INotuzumab Ozogamicin trial to inVestigAte Tolerability and Efficacy (INO-VATE) study, Kantarjian et al. demonstrated significant improvements in CR rates and OS compared to standard chemotherapy in relapsed or refractory B-cell ALL [30]. Side effects included hepatotoxicity and veno-occlusive disease (VOD), occurring in 11% of patients [30].

CAR T-cell therapy includes tisagenlecleucel and brexucabtagene autoleucel [31]. In a pivotal phase II trial (ELIANA), tisagenlecleucel achieved an overall remission rate of 81% and a 12-month event-free survival (EFS) rate of 50% in pediatric and young populations with relapsed or refractory B-ALL [32]. Severe side effects include CRS, observed in 77% of patients, and neurotoxicity, occurring in 40% of patients. Long-term follow-up data demonstrate sustained remissions in a subset of patients [32]. In adult patients, brexucabtagene autoleucel is approved for relapsed or refractory B-cell ALL [32]. This therapy targets CD19, leading to significant response rates [32]. In the ZUMA-3 trial, Brexu-cel achieved a CR rate of 68% with a median duration of response of 12.8 months. Side effects include CRS (91%) and neurotoxicity (70%) [32].

A combination therapy of blinatumomab and dasatinib has shown promise in treating Philadelphia chromosome-positive (Ph+) B-ALL [33]. In a phase II trial by Foà et al., the combination achieved a three-year OS rate of 95% in newly diagnosed Ph+ B-ALL patients [34]. Blinatumomab, targeting CD19, is the first BiTE approved for relapsed or refractory B-ALL, showing superior efficacy compared to chemotherapy in phase III trials [35]. Novel BiTEs targeting CD20 and CD22 are being developed [36].

Checkpoint inhibitors that target PD-1, PD-L1, and CTLA-4 are being explored with other ALL immunotherapies and chemotherapy [37]. Natural killer (NK) cell therapy is a form of adoptive cell therapy that uses NK cells, which can recognize and kill abnormal cells without prior sensitization, and has demonstrated encouraging outcomes in specific individuals experiencing recurring or resistant cases of ALL or AML [31]. Dendritic cell (DC) therapy is a form of active immunotherapy that uses DCs to activate T cells and induce antitumor immunity [31]. It has shown some efficacy and safety in individuals with recurring or resistant cases of ALL or AML who received DCs loaded with leukemia antigens [32]. Tumor vaccines are another form of active immunotherapy that uses tumor antigens or whole tumor cells to stimulate the immune system against leukemia cells and have shown some efficacy and safety in children with recurring or resistant cases of ALL or AML who received vaccines derived from autologous or allogeneic leukemia cells [33].

Oncolytic viruses target and eliminate cancer cells without harming healthy cells, have shown some antileukemic activity and safety in preclinical models, and are now being considered in trials for various cancers [34]. However, these novel immunotherapies face safety issues, manufacturing difficulties, regulatory hurdles, and cost-effectiveness challenges [35]. Safety issues include the potential for off-target effects, immunogenicity, cytokine storm, GVHD, or viral reactivation [36]. Manufacturing difficulties include the complexity, variability, and scalability of personalized or standardized immunotherapy products [37]. Regulatory hurdles include clear guidelines, standards, and criteria for developing, approving, and monitoring immunotherapy products [38]. Cost-effectiveness involves evaluating the economic value and impact of immunotherapy products compared to other therapies [39].

Immunotherapy has substantially improved the cure of leukemia, offering hope for patients with relapsed or refractory disease. Despite promising clinical outcomes, challenges such as resistance, toxicity, and accessibility must be addressed. Current projects continue to explore novel combinations and therapeutic approaches to improve patient outcomes and reduce adverse effects.

Advancements in chemotherapy regimens for pediatric leukemia: enhancing efficacy and managing relapse

Chemotherapy for ALL in children is intensive, complex, and prolonged, with optimal outcomes associated with adherence to contemporary research protocols [40]. Therapy for ALL involves several phases, each targeting specific goals to ensure comprehensive disease management [40]. The main phases include management of the CNS, remission induction, consolidation/late intensification, and maintenance [41]. Chemotherapy regimens in pediatric ALL differ significantly from those used in adult ALL due to age-specific considerations and treatment goals [41,42]. Pediatric protocols typically aim for high cure rates while minimizing long-term toxicity [41,42]. These regimens often incorporate higher doses of cytotoxic agents and longer durations of maintenance therapy than adults [43,44].

Additionally, pediatric protocols may include CNS-directed therapies to prevent leukemia from spreading to the brain and spinal cord, reflecting the higher incidence of CNS involvement in pediatric ALL [44,45]. In contrast, chemotherapy regimens for adult ALL are generally more intensive initially, focusing on achieving CR quickly due to the disease's higher aggressiveness in adults [45,46]. Consolidation therapies may vary, including allogeneic hematopoietic SCT for suitable candidates to reduce relapse risk [45,46]. Maintenance therapy in adults is typically shorter and less intensive than pediatric protocols, aiming to balance efficacy with minimizing long-term side effects [45,46]. This distinction underscores the importance of age-specific treatment approaches in ALL, tailored to optimize outcomes while considering the differing biological characteristics and tolerances between pediatric and adult populations [41-45].

CNS involvement is a critical concern in ALL treatment, necessitating both prophylactic and therapeutic measures to prevent relapse [41]. All patients receive CNS prophylaxis to reduce relapse risk [41]. Methods vary among protocols, often involving intrathecal (IT) chemotherapy with agents such as cytarabine, methotrexate, and hydrocortisone [41]. Cranial RT is generally avoided due to the risk of late adverse effects like cognitive deficits and secondary malignancies [41]. Patients with confirmed CNS involvement require augmented CNS-directed therapy, including frequent IT chemotherapy and high-dose systemic chemotherapy (e.g., high-dose cytarabine) [41]. Cranial RT is usually avoided to minimize late AEs [40,41].

Remission induction aims to reduce the disease burden, achieve CR, and restore normal hematopoiesis [41]. Therapy is stratified based on clinical and pathological features at presentation, involving multiagent chemotherapy regimens tailored to the patient profile [41]. Unique approaches are required for subtypes like T-cell ALL, Philadelphia chromosome-positive (Ph+) ALL, and Ph-like ALL [41,42].

Consolidation therapy aims to deepen remission and reduce relapse risk, while late intensification further eradicates residual disease [43]. Low-risk patients typically receive high-dose cytarabine-based chemotherapy [43]. The options for intermediate/high-risk patients include high-dose cytarabine-based chemotherapy or allogeneic hematopoietic SCT, depending upon individual risk factors and treatment responses [43]. Maintenance therapy, consisting of lower-intensity chemotherapy, spans two to three years to sustain remission [43]. Common agents include mercaptopurine and methotrexate [43]. Emerging strategies are being explored to enhance long-term outcomes and reduce toxicity [41-43].

CNS therapy is tailored based on the risk of CNS relapse [41]. High-risk factors include leukemic cells in the initial lumbar puncture, high leukocyte counts at diagnosis, T-cell phenotype, and specific genetic markers like the Philadelphia chromosome [41]. Conversely, B-cell ALL without these characteristics is considered lower risk [41]. For high-risk patients, CNS-directed therapy typically involves IT treatments, systemic therapy, and sometimes cranial RT [41]. IT therapy may include methotrexate or a combination of methotrexate, cytarabine, and hydrocortisone alongside high-dose methotrexate and increased doses of L-asparaginase [41,42]. The use of cranial RT is debated and often reserved for those at the highest risk of CNS relapse [41]. Lower-risk patients usually receive IT therapy as prophylaxis instead of cranial RT due to its associated adverse effects [41].

The first IT treatment coincides with the initial diagnostic lumbar puncture [41]. Systemic therapy intensification is crucial for reducing relapse in high-risk patients and must align with the selected treatment protocol [41-43]. Contemporary treatments have reduced CNS relapse to less than 5% in children achieving complete CR [42]. IT therapy has shown a low rate of CNS relapse and acceptable toxicity levels [42]. In contrast, cranial RT, while reducing CNS relapse rates, is linked to significant long-term morbidity, particularly in younger children [42].

Systemic therapy for pediatric ALL is divided into several phases, each with specific agents and strategies based on risk stratification [43]. Induction therapy includes a glucocorticoid, vincristine, and asparaginase, with some protocols adding an anthracycline [43]. Post-induction therapy is guided by the patient's response, with MRD assessment playing a pivotal role [44]. Maintenance therapy involves less intensive outpatient chemotherapy with ongoing CNS therapy, typically lasting two to two and a half years [44]. Adherence to maintenance therapy is crucial, as even slight deviations can significantly impact relapse rates [44].

Chemotherapy remains the cornerstone of ALL treatment [15]. Induction chemotherapy comprises a glucocorticoid, vincristine, and asparaginase [45]. Dexamethasone, with better CNS penetration, is often chosen over prednisone for higher-risk cases despite its higher adverse effect profile [46]. Chemotherapy involves using powerful anti-cancer drugs to eliminate or halt the growth of leukemia cells [47]. Its fundamental aims include destroying leukemia cells in the blood and bone marrow, attaining and maintaining remission, and preventing leukemia cell spread to the brain and spinal cord [47,48]. The selection and dosage of drugs are influenced by varying factors like leukemia type (ALL or AML), risk category, treatment response, and adverse effects [48].

Several chemotherapy medications are utilized to treat pediatric leukemia, including vincristine, daunorubicin, doxorubicin, and cytarabine, among others [49]. Children undergoing chemotherapy for leukemia are at risk of various acute and long-term side effects, necessitating vigilant monitoring and comprehensive supportive care strategies to mitigate these outcomes [49]. Acute adverse reactions such as hair loss, mouth ulcers, gastrointestinal disturbances, and decreased blood cell count leading to infection susceptibility are managed through supportive medications, nutritional support, and infection prevention protocols during treatment phases [49,50]. Specialized interventions, such as growth hormone therapy for growth problems or fertility preservation measures for potential infertility, are crucial to address long-term effects [49,50]. Proactive, supportive care measures are essential to reduce and manage these effects [49,50]. Preemptive use of growth factors like granulocyte colony-stimulating factor (G-CSF) can help mitigate neutropenia-related infections [49,50]. At the same time, anti-emetics and mucosal protectants aid in minimizing gastrointestinal discomfort and mucositis [49,50]. Regularly monitoring kidney function and electrolyte levels during treatment for signs of tumor lysis syndrome is critical, with prompt intervention to prevent renal or cardiac complications [49,50]. Psychosocial support for children and families is equally vital to address the emotional and psychological challenges associated with treatment [49,50]. Educational support programs help manage potential learning difficulties from treatment-related cognitive impairments [49,50]. Long-term survivorship care plans, including monitoring secondary cancers and cardiovascular health, ensure timely detection and intervention for late effects, promoting optimal health outcomes into adulthood [50,51].

Recent studies underscore chemotherapy's effectiveness, revealing its benefits and associated side effects [51-55]. For ALL, chemotherapy is the mainstay treatment [51]. In pediatric patients, it is commonly administered in multiple phases [51]. Induction therapy aims to achieve remission, eliminating signs of leukemia from the body [52]. Drugs such as vincristine, corticosteroids, L-asparaginase, and anthracyclines are often used [52]. CNS prophylaxis prevents leukemia cells from spreading to the brain and spinal cord [53]. This may involve intrathecal chemotherapy or radiation to the head [53]. Consolidation therapy eradicates undetectable leukemia cells to prevent relapse [54]. High chemotherapy or stem cell transplant doses can be part of this phase [54]. Maintenance therapy involves lower doses of chemotherapy to maintain remission and lower the risk of relapse for an extended period, usually two to three years [55].

In AML, chemotherapy plays a critical role, though the regimens might differ from ALL [56]. Induction, consolidation, and maintenance phases are typical, but the specific drugs and dosages may vary [56]. Agents such as cytarabine, anthracyclines, and targeted therapies like GO are used [57]. A randomized Children's Oncology Group (COG) trial compared two different induction regimens for children with newly diagnosed standard-risk B-ALL [58]. The study found that adding a second dose of intrathecal methotrexate to the standard regimen of vincristine, dexamethasone, and PEG-asparaginase improved the event-free survival (EFS) and reduced the risk of CNS relapse without increasing toxicity [58].

In the International Berlin-Frankfurt-Münster Study Group (I-BFM-SG) Trial, Franco et al. examined various consolidation regimens for high-risk ALL in newly diagnosed children [59]. The phase 3 randomized trial, encompassing 3,202 children diagnosed with high-risk ALL between 2000 and 2006, compared two consolidation regimens: high-risk methotrexate (HR-MTX) and high-risk methotrexate plus mitoxantrone (HR-MTX+MITO) [59]. HR-MTX included four courses of high-dose methotrexate and asparaginase, while HR-MTX+MITO added two courses of mitoxantrone and cytarabine to the former regimen, followed by maintenance therapy with 6-mercaptopurine and methotrexate [59]. The primary outcome was the five-year EFS, with HR-MTX+MITO demonstrating a statistically significant advantage over HR-MTX (80.5% vs. 76.6%) [59]. Moreover, the secondary outcome of the five-year cumulative incidence of relapse favored HR-MTX+MITO, showing a reduction in the risk of relapse (14.2% vs. 18.4%) [59]. However, the five-year OS rates, though not statistically significant (86.8% vs. 83.9%), showed a trend favoring HR-MTX+MITO [59]. The study results indicate that incorporating mitoxantrone into the standard regimen notably enhanced EFS and reduced relapse risk without elevating toxicity for children with high-risk ALL [59]. The trial concluded that HR-MTX+MITO represents an improved consolidation regimen and could become the new standard of care for this patient group [59].

Another study involved a retrospective analysis investigating the outcomes of relapsed cases in children and adolescents with B-cell non-Hodgkin lymphoma (B-NHL) and mature acute leukemia (MAL) under the French LMB protocol from 2001 to 2011 [60]. Among 781 patients in the LMB2001 study, 69 experienced relapse [60]. The timing of relapse categorized patients: early (ER), within six months after treatment; late (LR), over six months post-treatment; and very late (VLR), over two years post-treatment [60]. The study focused on 5-year OS and secondary outcomes: 5-year EFS and cumulative incidence of second relapse (CIR) following a second complete remission (CR2) [60]. Results showed disparate 5-year OS rates -23 % for ER, 53% for LR, and 100% for VLR- reflecting significant differences among the groups [60]. EFS rates followed a similar pattern, with 19% for ER, 47% for LR, and 100% for VLR [60]. The 5-year CIR rates stood at 33% for ER, 13% for LR, and 0% for VLR [60]. The findings suggested unfavorable outcomes for early relapses, while later relapses had improved results, possibly influenced by rituximab use [60]. The study concluded that managing relapse in B-NHL and MAL in young pediatric patients remained challenging and necessitated novel strategies to prevent and treat relapse [60].

A meta-analysis by McGrath et al. examined the effectiveness and safety of adding GO to induction chemotherapy for patients with AML [61]. It involved 3,325 patients from five trials and compared outcomes between those receiving induction chemotherapy alone and those receiving it along with GO [61]. The addition of GO significantly improved OS (28.7% vs. 25.9%) and EFS (19.8% vs. 15.3%), primarily by reducing the risk of relapse, without substantially increasing overall toxicity, except for a higher risk of VOD [61]. Importantly, patients with certain factors, such as favorable or intermediate cytogenetic risk and CD33-positive AML, seemed to benefit more from GO [61]. The study suggested that GO be standard in induction chemotherapy for adult AML patients [61].

These studies demonstrate the significance of tailored chemotherapy regimens in different risk groups for pediatric ALL and AML [56-61]. They underscore that certain drug combinations or adjunct therapies may significantly improve survival rates, reduce the risk of relapse, and minimize toxic effects in children receiving chemotherapy for leukemia [56-61]. However, it is essential to consider potential side effects such as hair loss, nausea, and long-term complications, emphasizing the need for close monitoring and supportive care for these pediatric patients undergoing chemotherapy [56-61].

The critical role of BMT in pediatric leukemia: challenges and advances

BMT, or SCT, is a vital treatment modality for ALL and AML in pediatric patients [60]. This procedure involves replacing diseased or damaged bone marrow with healthy stem cells to restore the body's ability to produce blood cells and improve survival rates [60]. BMT is particularly beneficial for AML due to its high relapse rates and the presence of genetic mutations that increase the likelihood of cancer returning [60,61].

The typical BMT process for AML follows intensive chemotherapy designed to eradicate cancer cells before introducing healthy stem cells [62]. The most common type of transplant for AML is allogeneic SCT, wherein stem cells are sourced from a donor whose tissue type closely matches the patient's [62]. Autologous SCT, which uses the patient's stem cells, is less common due to the risk of reintroducing leukemia cells [62]. In the context of ALL, SCT enables the administration of higher doses of chemotherapy (and sometimes radiation) to eliminate cancer cells, followed by the infusion of blood-forming stem cells to replenish the patient's bone marrow [63]. SCT is often considered for ALL patients who are at high risk of relapse or have relapsed after initial therapy [63]. The decision to undergo a transplant is influenced by factors such as the patient's age, overall health, and specific leukemia characteristics [63].

BMT is also crucial in pediatric leukemia treatment, offering potentially better outcomes due to children's higher tolerance for high-dose chemotherapy and the robust regenerative capacity of their tissues [61]. However, BMT has significant risks, including GVHD, infections, and bleeding complications [64]. Recent advances like reduced-intensity conditioning (RIC) are being explored to decrease toxicity while maintaining efficacy [64]. Despite its life-saving potential, BMT remains a complex and risky procedure, necessitating individualized decisions [64]. 

Integrating personalized medicine, which tailors treatment decisions to each patient's unique genetic profile and disease characteristics, is essential for improving BMT outcomes [64]. As research advances, more refined criteria for patient selection and improved supportive care measures are expected to enhance the procedure and efficacy [64].

There are different types of BMT based on the source of stem cells. In an autologous transplant, the child's cells are collected before intensive treatment, and after treatment, these preserved stem cells are reintroduced to generate new, healthy blood cells [62]. This type of transplant avoids the risk of rejection or GVHD but may not be effective if leukemia cells are present in the collected stem cells [62]. In an allogeneic transplant, healthy stem cells replace the damaged or cancerous bone marrow [63]. This approach provides a more substantial anti-leukemia effect due to the graft-versus-leukemia effect, where the immune cells attack any remaining leukemia cells [61-63]. However, it also carries a higher risk of complications, such as GVHD and infections [61-63].

BMT is often considered when other treatments, such as chemotherapy, fail to eradicate leukemia cells [60]. It is also used for rare leukemia forms, such as those with the Philadelphia chromosome or T-cell leukemia [62]. Before the transplant, the child undergoes high-dose chemotherapy and radiation therapy to destroy leukemia cells in the bone marrow [61-63]. Then, the donor's stem cells are infused into the child's bloodstream, where they migrate to the bone marrow and produce new blood cells [64]. 

Finding a suitable donor is critical for a successful transplant [63]. The donor should be a close genetic match to the recipient, often a sibling or an unrelated donor found through registries or cord blood banks [65]. The matching process can take weeks or months [65]. Before the transplant, patients receive high-dose chemotherapy and radiation therapy to destroy their existing bone marrow, making room for the donor's cells [66,67]. This conditioning regimen can cause severe side effects, including nausea, vomiting, hair loss, mouth sores, infections, bleeding, and organ damage [68]. Patients may need blood transfusions and antibiotics to support their health during this phase [69]. 

The transplant involves infusing the donor's stem cells into the patient's bloodstream, where they migrate to the bone marrow and produce new, healthy blood cells [69]. This process can take several weeks or months, during which the patient is vulnerable to infections and bleeding due to low blood cell levels [67-69]. Patients may also experience fever, chills, pain, swelling, or allergic reactions during or after the infusion [66-70]. 

Intensive medical care and monitoring are required post-transplant to manage complications such as GVHD, infections, and other side effects [62-64]. GVHD occurs when the immune cells attack the patient's healthy tissues and organs, causing skin irritation, gastrointestinal issues, liver complications, or respiratory problems [64,65]. GVHD can be acute (within 100 days after transplant) or chronic (after 100 days) [68]. It is treated with immunosuppressive drugs or other therapies [70]. Managing relapse in children who have undergone BMT for leukemia poses significant challenges despite advances in treatment modalities [71]. Relapse may occur due to residual leukemia cells that evade initial treatment, immune system failure to eradicate all malignant cells, or the emergence of therapy-resistant clones [71,72]. Current therapeutic approaches often include salvage chemotherapy regimens, donor lymphocyte infusion (DLI) to induce a graft-versus-leukemia effect, or second transplantation for eligible patients [71,72]. However, these strategies are associated with substantial risks, such as increased toxicity, GVHD, and limited efficacy in refractory cases [71,72]. Potential novel therapeutic strategies under investigation include BiTEs, CAR T-cell Therapy, targeted therapies, and immune checkpoint inhibitors, as discussed previously [71,72]. Regular blood tests, imaging tests, biopsies, or other procedures are necessary to check blood counts, organ function, and signs of relapse or infection [70]. Patients may also need vaccinations, nutritional supplements, physical therapy, psychological support, or other services to aid their recovery and adjustment to a new life [69-71].

A team of healthcare professionals decides to proceed with a BMT based on the patient's specific circumstances and donor availability [70-72]. The study by Panuciak et al. was a prospective trial of myeloablative haploidentical BMT (haplo-BMT) with posttransplant cyclophosphamide for pediatric acute leukemias [73]. The study enrolled 25 patients with relapsed or refractory ALL or AML who received haplo-BMT from a partially matched family donor [73]. The study reported that haplo-BMT with posttransplant cyclophosphamide was a safe and feasible option for pediatric patients with high-risk acute leukemias [73]. The study achieved 0% transplant-related mortality and 0% grade III or IV acute GVHD in the patients [73]. The study also showed a two-year OS of 64% and a two-year event-free survival (EFS) of 56% in the patients [73]. The study concluded that haplo-BMT with posttransplant cyclophosphamide could be a promising alternative for pediatric patients who lack a matched donor [73].

A retrospective analysis examined the impact of disease risk on the efficacy of matched sibling donor BMT versus chemotherapy alone for pediatric AML in the first CR [74]. This study compared the outcomes of 1,373 pediatric patients with AML in first CR, with 555 receiving BMT and 818 receiving chemotherapy alone [74]. The study found that BMT was associated with improved OS and relapse-free survival in patients with high-risk or intermediate-risk disease but not in patients with low-risk disease [74].

The study by Tsang aimed to provide evidence-based recommendations for immunization schedules, vaccine types, and contraindications for pediatric BMT recipients [75]. The study found a lack of consensus and standardization among the immunization guidelines for pediatric BMT recipients [75]. The immunization response and safety varied depending on the vaccine type, the timing of administration, and the patient's immune status [75].

## Conclusions

In conclusion, the rapid strides in pediatric leukemia treatments, including immunotherapy, monoclonal antibodies, and BMT, herald a promising era of improved outcomes and enhanced survival for young patients. The remarkable progress in these innovative therapies signifies a monumental leap forward in managing this formidable disease. However, the journey towards consistently better outcomes requires continuous research, collaborative endeavors, and the seamless integration of these novel approaches into standard care. It is essential to recognize and address the difficulties and limitations linked with these advanced therapies, thereby enhancing their efficacy and safety profiles. As we navigate these therapeutic horizons, personalized medicine will be increasingly crucial in tailoring treatments to individual patient needs, further optimizing outcomes.

The treatment of pediatric leukemia has advanced significantly with the integration of immunotherapy, chemotherapy, and BMT, offering varied therapeutic options depending on disease subtype and risk factors. Immunotherapy, including monoclonal antibodies and CAR T-cell therapy, has shown remarkable efficacy in treating relapsed or refractory cases, providing new avenues for remission, and improving survival outcomes. Chemotherapy remains a cornerstone in treatment protocols, with tailored regimens enhancing remission rates and minimizing toxicity in pediatric patients. Allogeneic SCT, especially in high-risk or relapsed cases, offers a potential cure despite inherent risks such as GVHD and infections. However, challenges persist, including treatment resistance, treatment-related toxicity, and disparities in access to innovative therapies. Ethical considerations in pediatric leukemia treatment are crucial, encompassing issues such as informed consent, balancing treatment risks with potential benefits, and ensuring equitable access to cutting-edge therapies across diverse socioeconomic backgrounds. Decision-making regarding intensive treatments like BMT involves carefully considering long-term quality-of-life impacts, particularly concerning potential late effects such as secondary malignancies or organ dysfunction.

To address these ethical challenges, ongoing research must prioritize patient-centered outcomes, integrate ethical frameworks into clinical trials, and enhance transparency in treatment decision processes. Collaborative efforts among healthcare providers, researchers, and patient advocacy groups are essential to promote ethical guidelines and ensure that treatment advancements are accessible and equitable for all pediatric leukemia patients.
